# Neurological implications of Multisystem inflammatory syndrome in children (MIS-C) in African settings

**DOI:** 10.11604/pamj.2025.52.9.49094

**Published:** 2025-09-10

**Authors:** Daniel Matovu

**Affiliations:** 1 Department of Neurology and Clinical Pharmacology, School of Medicine, Kabale University, Kabale, Uganda

**Keywords:** Multisystem inflammatory syndrome in children (MIS-C), COVID-19, neurological complications, sub-Saharan Africa, pediatric neurology

## To the editors of the Pan African Medical Journal

We have read the recent case report by Ebrahim *et al*. in The Pan African Medical Journal (2025; 51: 96) vividly illustrating the challenges of diagnosing and managing multisystem inflammatory syndrome in children (MIS-C) associated with COVID-19 in African contexts [1]. While the report emphasizes cardiac and gastrointestinal manifestations, the neurological implications of MIS-C remain underexplored, particularly in sub-Saharan Africa, where diagnostic resources are scarce. As a brain clinical researcher at Kabale University School of Medicine, we advocate for urgent research into MIS-C´s neurological sequelae to enhance clinical outcomes in resource-limited settings. Multisystem inflammatory syndrome in children, a post-infectious hyperinflammatory condition linked to SARS-CoV-2, typically presents with fever, multisystem involvement, and elevated inflammatory markers such as C-reactive protein and ferritin [2]. Globally, neurological complications, including encephalopathy, seizures, and stroke, occur in approximately 20% of MIS-C cases [3]. However, in African settings, these manifestations are likely underdiagnosed due to limited access to neuroimaging and electroencephalography. Ebrahim *et al*. case, which describes a child with fever and abdominal pain, does not explicitly address neurological symptoms, potentially reflecting diagnostic gaps in the region [1]. In our practice in Uganda, we have observed children with MIS-C exhibiting subtle neurological signs-such as irritability, lethargy, or altered consciousness-that are often misattributed to common conditions like malaria or meningitis.

This letter introduces a novel perspective by emphasizing MIS-C´s neurological burden in Africa. Emerging evidence suggests that SARS-CoV-2 may trigger neuroinflammation through cytokine storms, potentially leading to long-term cognitive deficits [4]. In African children, who face high rates of malnutrition and coexisting infections, these effects may be exacerbated, resulting in worse outcomes. For instance, a South African study noted delayed MIS-C diagnoses due to symptom overlap with tuberculosis, a challenge likely compounded for neurological presentations [5]. At Kabale University Hospital, we recently encountered a 9-year-old with MIS-C who presented with seizures, initially misdiagnosed as cerebral malaria. This case highlights the critical need for region-specific diagnostic approaches that prioritize neurological assessment. To address this gap, we propose integrating simple, cost-effective neurological screening tools into MIS-C diagnostic protocols in African hospitals. Tools like the Glasgow Coma Scale or pediatric seizure checklists could identify early neurological involvement. Additionally, point-of-care tests for inflammatory markers could expedite diagnosis in settings with limited laboratory infrastructure. Training primary care providers to recognize subtle signs, such as focal deficits or behavioral changes, is essential, given the scarcity of neurologists in Africa. Collaborative research across African institutions, facilitated by platforms like PAMJ, could standardize data collection on MIS-C´s neurological impact, informing tailored clinical guidelines. Ebrahim *et al*. case report is a vital contribution to understanding MIS-C in Africa, but its neurological dimensions demand further exploration. By focusing on this understudied aspect, we can improve diagnostic accuracy and mitigate long-term sequelae in African children. We call on researchers and clinicians to investigate MIS-C´s neurological implications and develop resource-appropriate strategies to address this emerging challenge.
